# Identification of biomarkers associated with ferroptosis in macrophages infected with *Mycobacterium abscessus* using bioinformatic tools

**DOI:** 10.1371/journal.pone.0314114

**Published:** 2025-01-10

**Authors:** Jiahua Qian, Chenghua Lu, Kai Meng, Zhihong Xu, Honghao Xue, Weijie Yang

**Affiliations:** 1 Department of Respiratory Medicine, Putuo Hospital, Shanghai University of Traditional Chinese Medicine, Shanghai, China; 2 Department of Respiratory Medicine, Longhua Hospital, Shanghai University of Traditional Chinese Medicine, Shanghai, China; 3 Department of Traditional Chinese Medicine, Xuhui District Central Hospital, Shanghai, China; 4 Department of Geriatrics, Ruijin Hospital, Shanghai Jiao Tong University Medical College, Shanghai, China; 5 Department of Traditional Chinese Medicine, Ruijin Hospital, Shanghai Jiao Tong University Medical College, Shanghai, China; Fujian Provincial Hospital, CHINA

## Abstract

*Mycobacterium abscessus* is a rapidly growing nontuberculous mycobacterium that causes severe pulmonary infections. Recent studies indicate that ferroptosis may play a critical role in the pathogenesis of *M*. *abscessus* pulmonary disease. We obtained gene expression microarray data from the Gene Expression Omnibus database, focusing on THP-1-derived macrophages infected with *M*. *abscessus* and uninfected controls. Differentially expressed genes related to ferroptosis were identified through weighted gene co-expression network analysis and the "limma" package, followed by gene set variation analysis and gene set enrichment analysis for enrichment assessment. To explore regulatory network relationships among hub genes, we constructed RBP-mRNA, ceRNA, and TF-mRNA networks. Additionally, a protein-protein interaction network was built, and functional enrichment analyses were conducted for the hub genes. The diagnostic value of these genes was assessed using receiver operating characteristic curves. Six differentially expressed genes associated with ferroptosis were identified in *M*. *abscessus* infection. The receiver operating characteristic curves demonstrated that these genes had excellent predictive value for the infection. Functional enrichment analysis showed that these genes were involved in immune responses, inflammation, cellular metabolism, cell death, and apoptosis. Pathway enrichment analysis revealed significant enrichment in pathways related to apoptosis, inflammation, and hypoxia. The RBP-mRNA network highlighted significant interactions between hub genes and key RNA-binding proteins, while the ceRNA network predicted that miRNAs and lncRNAs regulate ferroptosis-related genes *NACC2* and *ITPKB*. Furthermore, interactions between the hub gene *HSD3B7* and transcription factors LMNB1 and ASCL1 may promote ferroptosis in macrophages by influencing iron metabolism and reactive oxygen species production, contributing to the *M*. *abscessus* infection process. Our findings identified biomarkers linked to ferroptosis in *M*. *abscessus* infection, providing new insights into its pathogenic mechanisms and potential therapeutic strategies.

## 1 Introduction

Nontuberculous mycobacteria (NTM) refer to a diverse group of mycobacteria that exclude the *Mycobacterium tuberculosis* complex and *Mycobacterium leprae* [1]. These infections are becoming increasingly common worldwide, sometimes even surpassing new tuberculosis cases in developed countries [2, 3]. NTM are ubiquitous in the environment, particularly in water, soil, and dust, and can infect various organs and tissues. Nontuberculous mycobacterial pulmonary disease (NTM-PD) is the most common form [4], with *M*. *abscessus* (MAB) being one of the predominant species. MAB is known for causing severe respiratory infections, especially in individuals with chronic diseases or compromised immune systems [5]. Its high level of antibiotic resistance presents significant treatment challenges [6]. MAB pulmonary disease (MAB-PD) develops gradually and presents symptoms like coughing and fatigue. Diagnosis often involves high-resolution CT scans, revealing distinctive features like cylindrical bronchiectasis and small nodules [7, 8]. Treatment remains difficult due to MAB’s resistance to many antibiotics, including macrolides and rifampin [6]. Current guidelines lack clear recommendations on the best treatment approaches [9]. Thus, the identification of genes with diagnostic significance for MAB infection is imperative, aiding in the development of novel therapeutic approaches.

Ferroptosis, a type of cell death, is marked by the oxidation of cell membrane lipids. It is induced by factors such as iron accumulation, lipid peroxidase activity, glutathione depletion, mitochondrial contraction, and increased membrane density [10]. In eukaryotes, ferroptosis is precisely regulated by iron metabolism and lipid peroxidation [11]. Ferrous iron and polysulfides can synergistically induce bacteria to undergo ferroptosis-like cell death [12]. In recent years, the incidence of chronic pulmonary infections caused by MAB has been increasing annually, particularly among immunocompromised patients [6, 13]. MAB establishes and maintains infections in the host through pathogenic mechanisms such as environmental adaptability, morphological changes, biofilm formation, genetic diversity, and multidrug resistance, which pose significant challenges to treatment [14]. Studies have shown that altering iron metabolism can induce ferroptosis-like cell death, enhancing the bactericidal effects of antibiotics through oxidative stress and leading to the eradication of MAB [15]. However, these studies are limited in scope, as they fail to deeply explore the molecular regulation of virulence factors and adaptive mechanisms. As a result, they do not provide a comprehensive understanding of MAB infection. Current research is insufficient because it does not fully consider the influence of host background and environmental factors. With the rising infection rate, there is an urgent need for more comprehensive mechanistic studies, particularly using multi-omics approaches to elucidate the pathogenic mechanisms of MAB. Moreover, no research to date has investigated biomarkers related to ferroptosis in MAB infections. Targeting ferroptosis could be an effective treatment strategy for MAB infection. We utilized bioinformatic tools to identify biomarkers associated with ferroptosis in MAB infection. This study aims to shed light on the mechanism of ferroptosis in MAB infection and improve its diagnosis and treatment.

## 2 Materials and methods

### 2.1 Data acquisition

**Fig 1:**
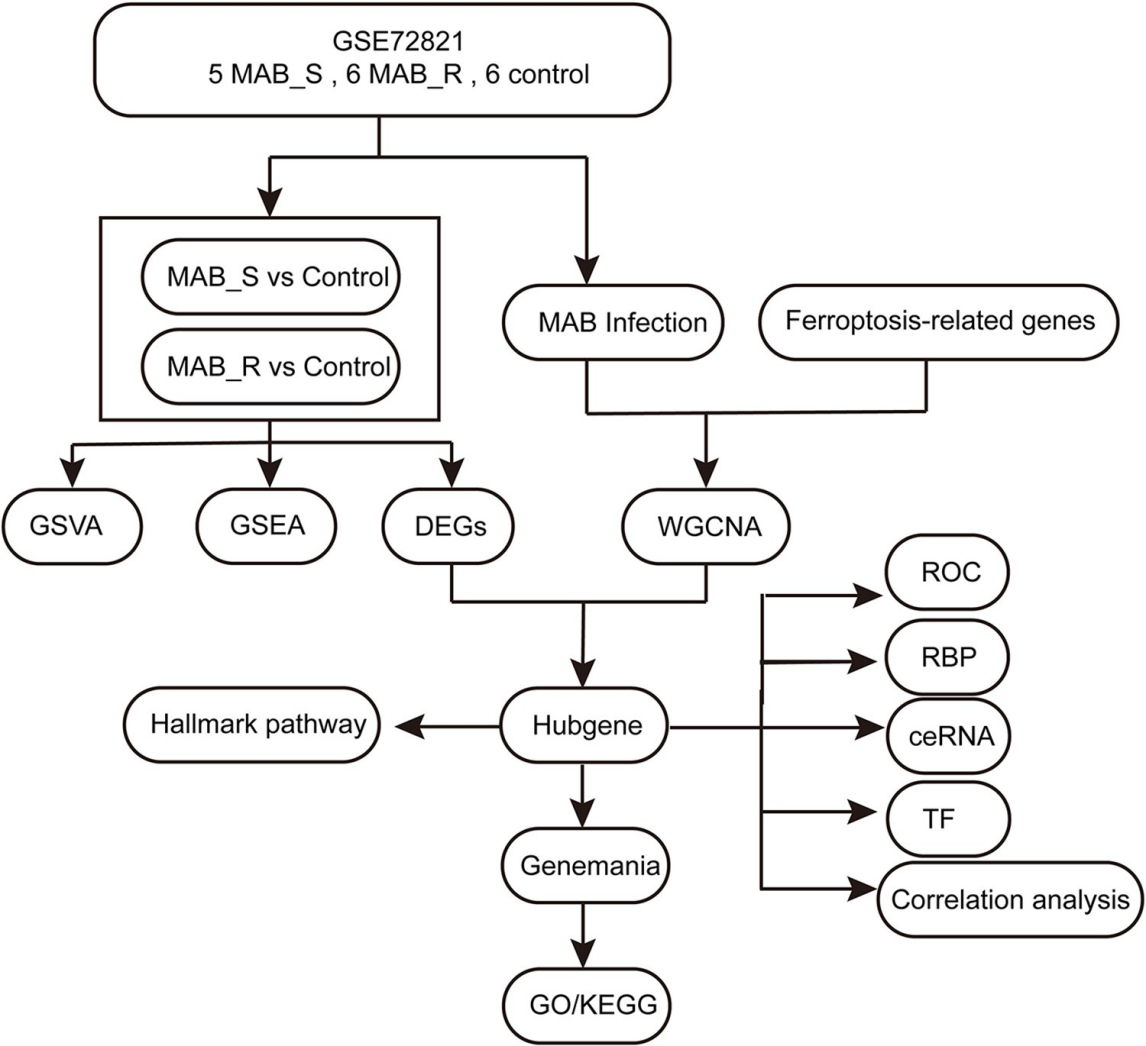
Overall scheme of this study. MAB_S: smooth-type Mycobacterium abscessus; MAB_R: rough-type Mycobacterium abscessus; GSVA: Gene Set Variation Analysis; GSEA: Gene Set Enrichment Analysis; DEGs: differentially expressed genes; WGCNA: Weighted Gene Co-expression Network Analysis; ROC: Receiver Operating Characteristic Curve; RBP: RNA binding protein; ceRNA: competing endogenous RNA; TF: Transcription factor; GO: Gene Ontology; KEGG: Kyoto Encyclopedia of Genes and Genomes.

All data used in this study were publicly sourced from the Gene Expression Omnibus (GEO) database (GEO, https://www.ncbi.nlm.nih.gov/geo/) database. The R package "GEOquery" was used to retrieve and download the GSE72821 dataset, which contains whole-genome expression profiles of MAB infection from the GEO database. The dataset included 17 samples, six samples were from THP-1-derived macrophages infected with rough-type MAB (MAB_R). Five samples were from THP-1-derived macrophages infected with smooth-type MAB (MAB_S). Furthermore, the dataset included six control samples. The GSM numbers of the samples are shown in Table 1. Information about 484 ferroptosis-related genes was obtained from the Ferroptosis Database V2 (FerrDb V2) (http://www.zhounan.org/ferrdb/current/) [16] (S1 Table). We downloaded ferroptosis driver genes, suppressor genes, and marker genes from this database. After merging and removing duplicates, we obtained a total of 484 genes representing ferroptosis. All data used in this study were obtained from publicly available databases, and their use adhered to the principles of the respective databases. Therefore, no additional ethical review was required. Fig 1 displays the layout of this study.

**Table 1:** GSM numbers of MAB infected THP-1-derived macrophages and control samples.

No.	GSM number	Sample category
1	GSM1873004	MAB_R
2	GSM1873005	MAB_R
3	GSM1873006	MAB_R
4	GSM1873007	MAB_R
5	GSM1873008	MAB_R
6	GSM1873009	MAB_R
7	GSM1873013	MAB_S
8	GSM1873014	MAB_S
9	GSM1873015	MAB_S
10	GSM1873016	MAB_S
11	GSM1873017	MAB_S
12	GSM1872995	Control
13	GSM1872996	Control
14	GSM1872997	Control
15	GSM1872998	Control
16	GSM1872999	Control
17	GSM1873000	Control

### 2.2 Analysis of differential gene expression in MAB infection

In this study, the R package "limma (version 3.50.0)" [17] was used to identify the genes differentially expressed among the control (n = 6), MAB_R (n = 6) and MAB_S groups (n = 5). The criteria for selection were |log2fold change| > 0.5 and p-values < 0.05, which were used for further analysis.

### 2.3 Gene set enrichment analysis (GSEA)

GSEA is a computational method designed to assess the consistency of predefined gene sets in showing significant differences among biological states [18]. Using the R package "clusterProfiler (version 4.2.2)" and GSEA, we ranked all genes based on their log2fold change values and conducted 1,000 gene set permutation analyses. In this study, "c2.cp.kegg.v7.5.1. symbols" from the Molecular Signatures Database (MSigDB) was selected as the reference gene set [18–20]. Gene sets with p-values < 0.05 were considered significantly enriched. In this study, we merged the control group (n = 6) with the six MAB_R samples (MAB_R/control) to create an expression matrix. Similarly, we merged the control group with five MAB_S samples to form an additional matrix (MAB_S/control). GSEA was performed separately for each group.

### 2.4 Gene set variation analysis (GSVA)

GSVA assesses associations between gene expression profiles and biological pathways or gene features without supervision or parameter assumptions [21]. To explore the differences in biological functions between the control and MAB infection groups, the "c2.cp.kegg.v7.5.1. symbols" gene set from the MSigDB database (http://software.broadinstitute.org/gsea/msigdb) was used as a reference. GSVA was performed using the R package "GSVA (version 1.42.0)". The results were visualized using the R package "pheatmap (version 1.0.12)". Fifty hallmark gene sets were downloaded as reference gene sets from the MSigDB database. The GSVA scores for each gene set across different samples were calculated using the single sample (ss)GSEA function in the GSVA package. Finally, the differences in GSVA scores for various gene sets between the control and MAB_R groups and between the control and MAB_S groups were compared using the “Limma” package.

### 2.5 Weighted gene co-expression network analysis (WGCNA) and identification of significant modules

The WGCNA algorithm was implemented using the R package WGCNA (version 1.70–3) to construct a co-expression network [22]. The similarity of gene expression profiles was assessed by calculating Pearson’s correlation coefficients, and the correlations between genes were weighted using a power function to construct a scale-free network. We used the "Pick Soft Threshold" function in the R package to set the power at β = 9, which helped construct a weighted adjacency matrix for co-expression similarity. Gene modules are dense clusters of co-expressed genes. WGCNA identifies these modules through hierarchical clustering, with different colors used to represent each module. We used a dynamic tree-cutting method to identify distinct modules. During module selection, we transformed the adjacency matrix, which measures topological similarity, into a topological overlap matrix (TOM). Module detection was then performed using clustering analysis. To assess the correlation between the modules and ferroptosis, Pearson’s correlation analysis was applied to calculate the module eigenvector (ME; the first principal component representing the overall expression of the module) in relation to ferroptosis. We identified modules significantly associated with ferroptosis. We visualized the structures of co-expression modules using a heatmap that shows the topological overlap of the gene network. The relationships among modules were summarized using hierarchical clustering of eigenvector networks and heatmaps. Finally, ferroptosis-related differentially expressed genes (DEGs) were identified by intersecting DEGs with the genes from ferroptosis-related modules.

### 2.6 Gene ontology (GO) pathway enrichment analysis

GO analysis is a commonly used method for large-scale functional enrichment studies covering biological processes (BP), molecular function (MF), and cellular components. The Kyoto Encyclopedia of Genes and Genomes (KEGG) is a widely used database that stores information on genomes, biological pathways, diseases, and drugs used to explore significantly altered metabolic pathways enriched in gene lists [23]. The R package "clusterProfiler (version 4.2.2)" was used for GO annotation and enrichment analysis of ferroptosis-related key genes (p-value < 0.05) [24].

### 2.7 GeneMANIA

The GeneMANIA website (http://genemania.org) helps predict the relationships between functionally similar genes and hub genes, encompassing protein-protein interactions (PPIs), protein-DNA interactions, pathways, physiological and biochemical responses, co-expression, and co-localization [25]. We used the GeneMANIA website to construct a PPI network of hub genes.

### 2.8 Receiver operating characteristic curve (ROC)

The ROC curve is an effective method for evaluating the diagnostic test performance. The ROC curve is a comprehensive indicator that reflects the sensitivity and specificity of continuous variables and visually depicts the interrelationship between sensitivity and specificity through graphical representation. The most common metric is the area under the curve (AUC) obtained from the ROC curve, which illustrates the sensitivity and specificity of the test. We used the R package "pROC" to create ROC curves and determine the AUC for selecting feature genes and assess their diagnostic value [26]. The area under the ROC curve generally falls between 0.5 and 1, with a higher AUC indicating better diagnostic performance. In this study, MAB-R and MAB-S samples were combined for MAB-PD (n = 11), and the AUC was calculated through comparison with the control group.

### 2.9 Construction of RBP-mRNA network

In this study, we utilized the widely used open-source platform StarBase (https://starbase.sysu.edu.cn/tutorialAPI.php#RBPTarget) to analyze RNA-RBP interactions. By employing CLIP-seq, degradome-seq, and RNA-RNA interaction data, we investigated associations between mRNA expression and RBPs (RNA binding proteins). In the context of disease, a significance threshold of p-value < 0.05, clusterNum ≥ 5, and clipExpNum ≥ 5 were established to define critical mRNA-RBP pairs. Subsequently, an RBP-mRNA network was constructed using the R package igraph.

### 2.10 Construction of ceRNA network

Given the ambiguous role of competing endogenous RNAs (ceRNAs) in the pathogenesis of MAB infection, we utilized the miRTarBase database (https://mirtarbase.cuhk.edu.cn/~miRTarBase/miRTarBase_2022/php/index.php) [27], StarBase2.0 (https://starbase.sysu.edu.cn/starbase2/index.php) [28], and miRDB (https://mirdb.org/index.html) to conduct reverse prediction of microRNAs (miRNAs) targeting hub genes, as well as to predict lncRNAs sharing common miRNAs with these hub genes. Ultimately, these efforts culminated in the construction of ceRNA network.

### 2.11 Construction of TF-mRNA network

Transcription factors (TFs) regulate the transcription process by recognizing specific DNA sequences, thus participating in various complex biological processes. The hTFtarget database (http://bioinfo.life.hust.edu.cn/hTFtarget#!) has compiled comprehensive TF target regulations from large-scale human transcription factor ChIP-Seq data, which includes 7,190 experimental samples from 659 transcription factors. These data encompass 569 conditions, including 399 cell lines, 129 tissue or cell types, and 141 treatment methods. By utilizing these data, it is possible to predict the common transcription factors of multiple genes.

### 2.12 Statistical analysis

Statistical analyses were conducted using R software version 4.1.2. Spearman’s correlation test was used to infer the correlation between two parameters. We used the Wilcoxon test for comparing two groups and the Kruskal-Wallis test for comparing three or more groups. Statistical significance was set at p < 0.05.

## 3 Results

### 3.1 Construction and module identification of weighted gene co-expression network

**Fig 2:**
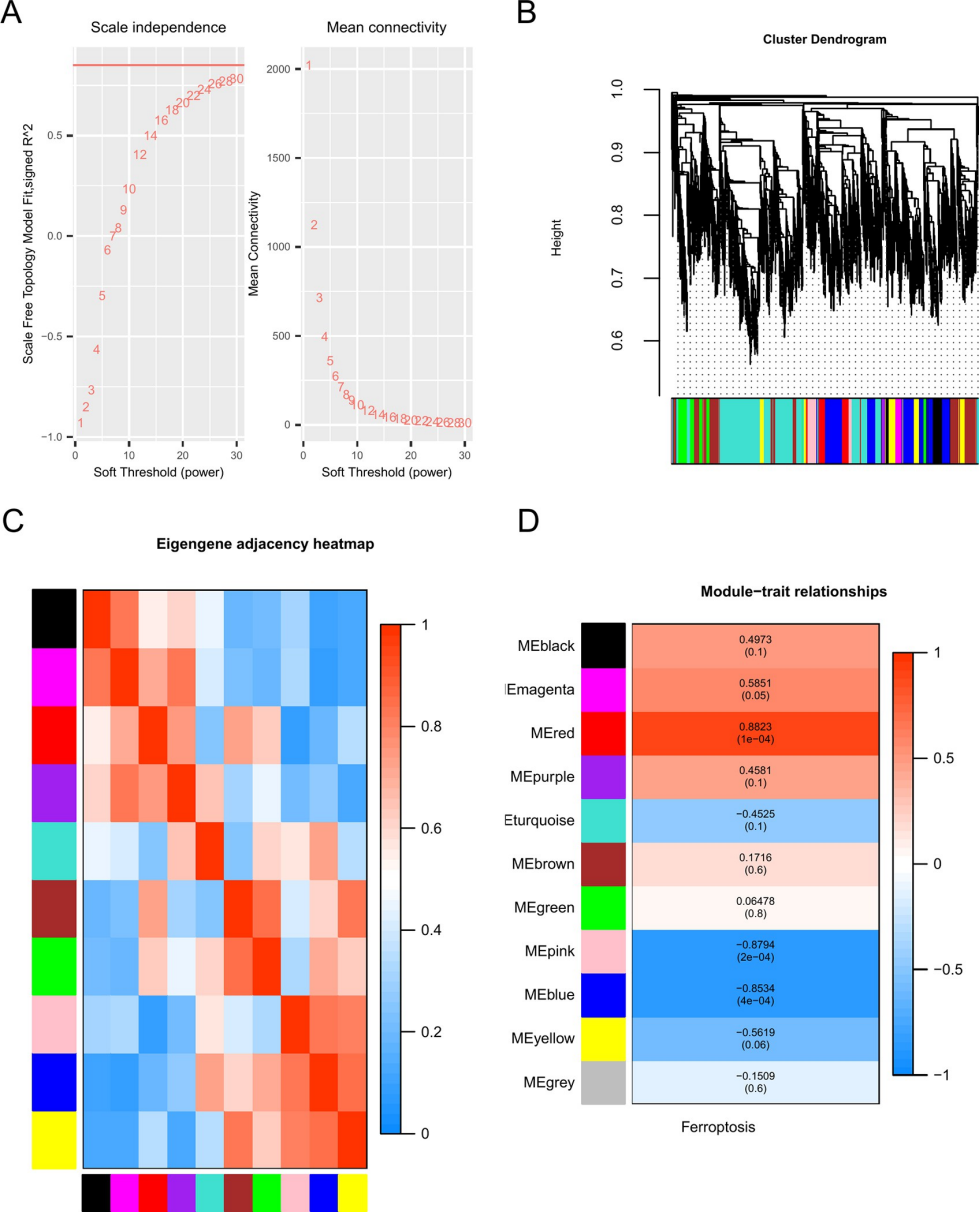
Construction of the weighted gene co-expression network analysis (WGCNA) co-expression network. (A) Soft threshold (β) = 9, Scale-free topology fit index (R^2). (B) Analysis of the gene expression network in THP-1-derived macrophages with *M*. *abscessus* infection revealed distinct modules of co-expressed data. (C) Relationship between modules depicted by a correlation heatmap of the feature gene network. Each row and column in the heatmap correspond to feature genes of a module (color-coded). In the heatmap, red indicates high adjacency, while blue indicates low adjacency. The red squares on the diagonal section represent the meta-modules. (D) Relationship between consistent module feature genes and ferroptosis. Each row in the table corresponds to a consistent module, and each column corresponds to a feature. The numbers in the table represent the correlation between the respective module feature genes and the trait, with the p-value below the correlation in parentheses. The color legend is used to encode the correlation according to the color.

We performed a WGCNA to investigate gene sets related to ferroptosis. The analysis showed that when the soft threshold (β) was set to 9 (Fig 2(A)), the average connectivity approached 0, and the scale independence was above 0.85. Ten co-expression modules were identified; unrelated genes were assigned to the grey module, which was ignored in subsequent studies (Fig 2(B)). To establish the relationships among modules, we correlated the ME with ferroptosis. We then created a dendrogram and heatmap to illustrate the feature gene network (Fig 2(C)). To understand the physiological significance of the genes within the modules, we correlated 10 MEs with ferroptosis and identified the most significant associations. Based on the heat map of the module-trait correlation (Fig 2(D)), we selected two modules with the highest positive and negative R values for ferroptosis-related phenotype genes. These modules included genes clustered in the pink module (n = 213, r = -0.8794, p < 0.05) and red module (n = 278, r = 0.8823, p < 0.05). We primarily considered the pink and red modules in subsequent analyses, as they were more likely to accurately indicate ferroptosis.

### 3.2 Differentially expressed genes in THP-1-derived macrophages infected with MAB_R

Upon comparing the samples of THP-1-derived macrophages infected with MAB_R with those of the control group, 1133 differentially expressed genes (DEGs) were identified; these genes exhibited statistically significant differences between the two groups (p < 0.05, |log2fold change| > 0.5). In MAB_R-infected macrophages, 340 genes were upregulated and 793 were downregulated (S2 Table). All DEGs were visualized using a volcano plot (S1(A) Fig). A heatmap illustrated the top five upregulated genes (*GPR157*, *KYNU*, *CXCL8*, *CXCL1*, and *CCL4*) and the top six downregulated genes (*RGS14*, *PLA2G15*, *CACFD1*, *OPRL1*, *CCDC121*, and *PNKP*) (S1(B) Fig). The rank-sum test confirmed significant differences in the expression of these 11 genes between the MAB_R samples and the control group (p < 0.05; S1(C) Fig).

### 3.3 GSEA of MAB_R samples

To explore the potential molecular mechanisms of DEGs, we performed GSEA. Using the MSigDB dataset, we selected signaling pathways with the most significant enrichment based on their normalized enrichment scores (NES) (S3 Table). GSEA results indicated significant enrichment in various pathways, including the chemokine signaling pathway (NES = 2.331, adjusted p = 0.046, FDR = 0.035), nod-like receptor signaling pathway (NES = 2.265, adjusted p = 0.039, FDR = 0.029), Janus kinase–signal transducer and activator of transcription (JAK-STAT) signaling pathway (NES = 2.258, adjusted p = 0.042, FDR = 0.032), pentose and glucuronate interconversions (NES = -1.634, adjusted p = 0.039, FDR = 0.029), drug metabolism cytochrome P450 (NES = -1.638, adjusted p = 0.039, FDR = 0.029), and retinol metabolism (NES = -1.709, adjusted p = 0.039, FDR = 0.029; S2(A)–S2(F) Fig). We performed GSVA to assess functional differences in pathway expression between the MAB_R group and the control group. The analysis enriched many pathways with distinct expression levels, which were visualized using a heatmap. Compared with the control group, the MAB_R group exhibited significantly low expression of KEGG_BASE_EXCISION_REPAIR and KEGG_BUTANOATE_METABOLISM and markedly high expression of the pathways associated with KEGG_CIRCADIAN_RHYTHM_MAMMAL and KEGG_NOD_LIKE_RECEPTOR_SIGNALING_PATHWAY (S2(G) Fig, S4 Table).

### 3.4 Differentially expressed genes in THP-1-derived macrophages infected with MAB_S

By comparing the samples of THP-1-derived macrophages with MAB_S and those of the control group, we identified 2882 differentially expressed genes (DEGs) with significantly different expression between the two groups (p < 0.05, |log2 fold change| > 0.5). In the samples of macrophages with MAB_S infections, 565 genes were upregulated and 2317 genes were downregulated (S5 Table). All DEGs were visualized using a volcano plot (S3(A) Fig). Additionally, a heatmap showed the top eight upregulated genes (*ZC3H12A*, *KDM6B*, *HIVEP2*, *IER3*, *SOCS3*, *IL1A*, *CCR7*, and *CCL4*) and the top five downregulated genes (*SPNS2*, *ARHGAP18*, *EVL*, *TBC1D14*, and *IVD*) (S3(B) Fig). Rank-sum tests further revealed significant differences in the expression of the top 13 genes between the MAB_S samples and the control group (p < 0.05; S3(C) Fig).

### 3.5 GSEA of MAB_S samples

To explore the potential molecular mechanisms of differentially expressed genes, we performed GSEA. Using the MSigDB dataset, we selected the most significantly enriched signaling pathways based on their normalized enrichment scores (NES) (S6 Table). GSEA results demonstrated significant enrichment in various pathways, including olfactory transduction (NES = -1.387, adjusted p = 0.032, FDR = 0.025), Huntington’s disease (NES = -1.478, adjusted p = 0.032, FDR = 0.025), oxidative phosphorylation (NES = -1.588, adjusted p = 0.032, FDR = 0.025), peroxisome (NES = -1.623, adjusted p = 0.032, FDR = 0.025), drug metabolism cytochrome P450 (NES = -1.653, adjusted p = 0.032, FDR = 0.025), and retinol metabolism (NES = -1.661, adjusted p = 0.032, FDR = 0.025; S4(A)-S4(F) Fig).To explore the functional annotations of MAB_S, we conducted GSVA to assess the differences in the relative expression of pathways between the two groups. GSVA enriched many pathways with different expression levels, which were visualized using a heatmap. Compared to that in the control group, the expression of KEGG_BASE_EXCISION_REPAIR and KEGG_PEROXISOME was significantly low and that of pathways related to KEGG_ACUTE_MYELOID_LEUKEMIA and KEGG_CYTOKINE_CYTOKINE_RECEPTOR_INTERACTION was significantly high in the MAB_S group (S4(G) Fig, S7 Table).

### 3.6 GO pathway enrichment analysis

**Fig 3:**
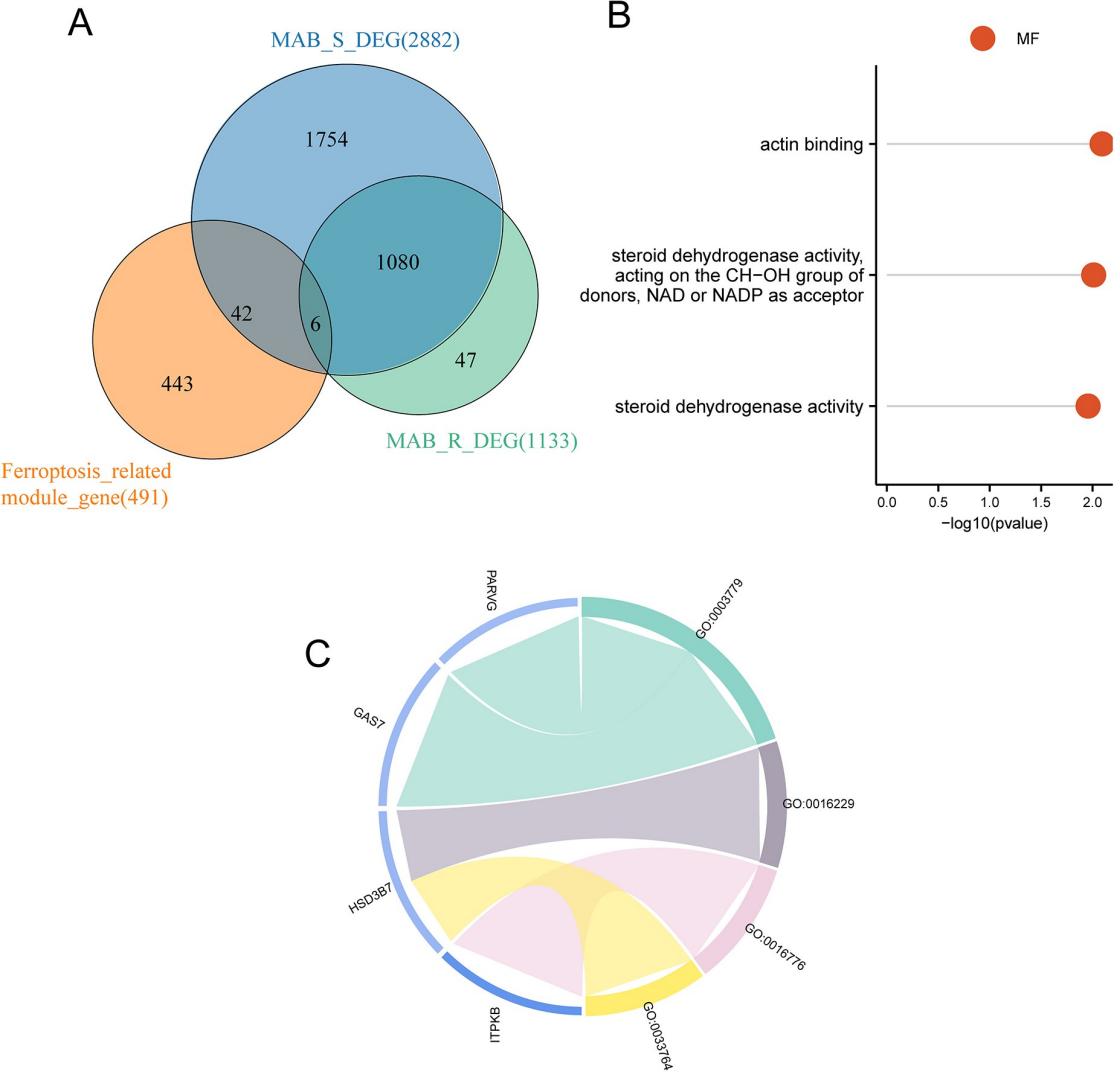
Enrichment analysis based on ferroptosis-related differentially expressed genes (DEGs). (A) Intersection of DEGs in MAB_S/Control, MAB_R/Control, and Ferroptosis-related module genes. (B) Lollipop chart showing gene ontology (GO) enrichment results. (C) Chord chart displaying GO enrichment results.

The intersection of differentially expressed genes (DEGs) between MAB_S/Control and MAB_R/Control resulted in 1086 intersected genes (Fig 3(A)). The intersection of these 1086 genes with key module genes from WGCNA yielded six hub genes (*GBP5*, *ITPKB*, *GAS7*, *NACC2*, *HSD3B7*, and *PARVG*) for further analysis. To investigate the biological functions of the ferroptosis-related DEGs, we conducted GO enrichment analysis on the six hub genes (S8 Table). The results indicated that these genes were enriched in actin binding (GO:0003779), steroid dehydrogenase activity acting on the CH-OH group of donors, NAD or NADP acting as acceptors (GO:0033764), and steroid dehydrogenase activity (GO:0016229; MF) (Fig 3(B) and 3(C)).

### 3.7 Expression patterns of hub genes

**Fig 4:**
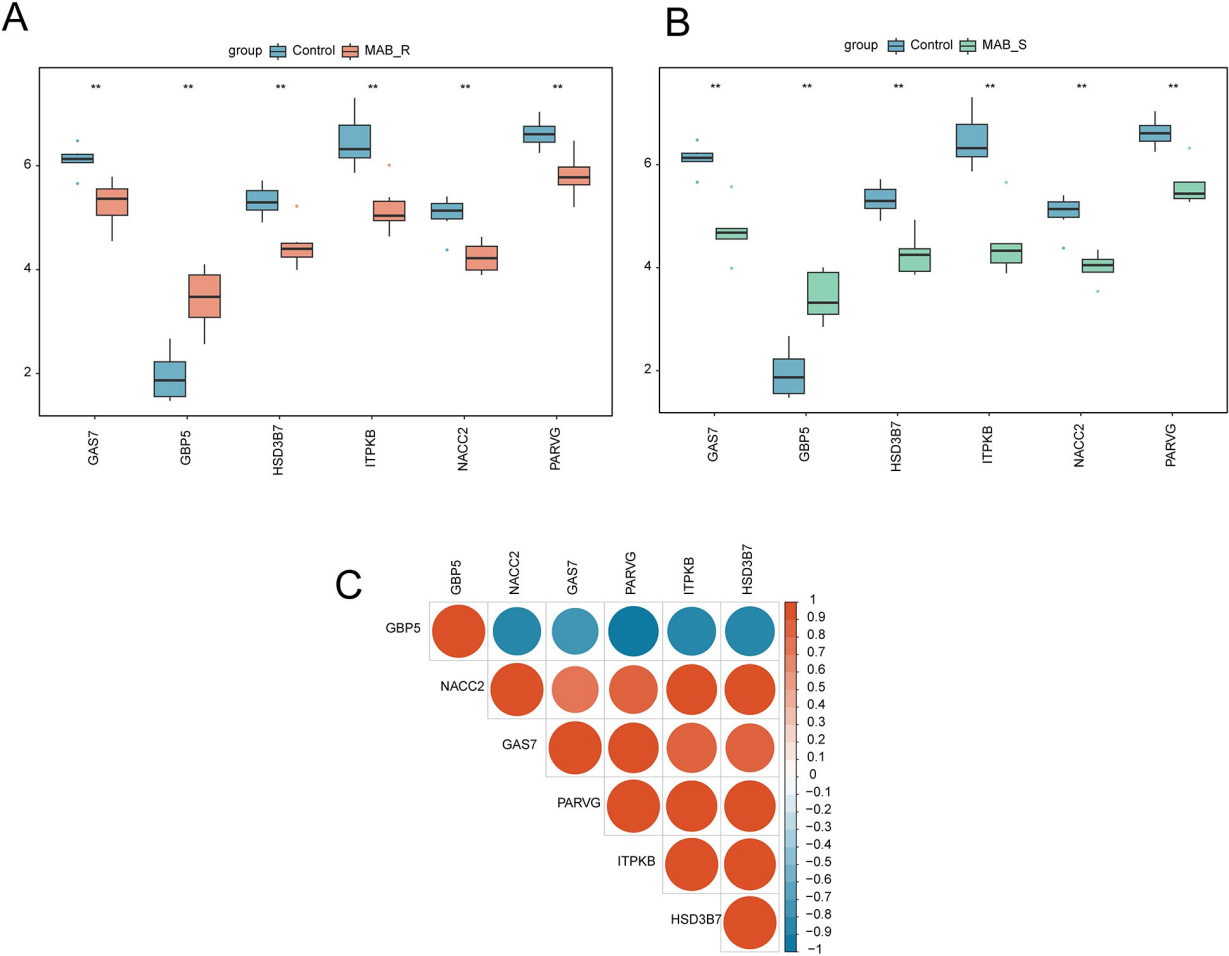
Expression patterns of hub genes. (A) Box plots depict variations in hub gene expression levels between MAB_R samples and the control group. (B) Box plots illustrate disparities in hub gene expression levels between MAB_S samples and the control group. (C) Correlation heatmap of hub genes. Significance is evaluated using the rank sum test. Asterisks represent p values: **** p < 0.0001, *** p < 0.001, ** p < 0.01, * p < 0.05.

We examined the expression profiles of hub genes in MAB_R, MAB_S, and the control group through box plots and correlation heatmaps. Notably, hub gene *GBP5* displayed markedly elevated expression levels in both MAB_R and MAB_S compared to the control group, while the expression of the remaining hub genes exhibited a significant decrease in both MAB_R and MAB_S (Fig 4(A) and 4(B)). Furthermore, *GBP5* showed a negative correlation with the other five hub genes, while the remaining hub genes were positively correlated (Fig 4(C)).

### 3.8 Hub gene interaction analysis

We used the GeneMANIA database to construct a PPI network for the hub genes, which revealed the interactions among the six genes (S5(A) Fig). To gain a deeper insight into the functions of these feature genes, we performed GO analysis on 26 genes, including the six hub genes and 20 genes associated with the hub genes. The GO enrichment analysis results revealed that these genes were enriched in interleukin-1 beta production (GO:0032611), regulation of interleukin-1 beta production (GO:0032651), interleukin-1 production (GO:0032612), regulation of interleukin-1 production (GO:0032652) (BP), pattern recognition receptor activity (GO:0038187), deaminase activity (GO:0019239), and hydrolase activity, acting on carbon-nitrogen (but not peptide) bonds, in cyclic amidines (GO:0016814; MF) (S5(B) Fig, S9 Table).

### 3.9 Diagnostic value of hub genes

**Fig 5:**
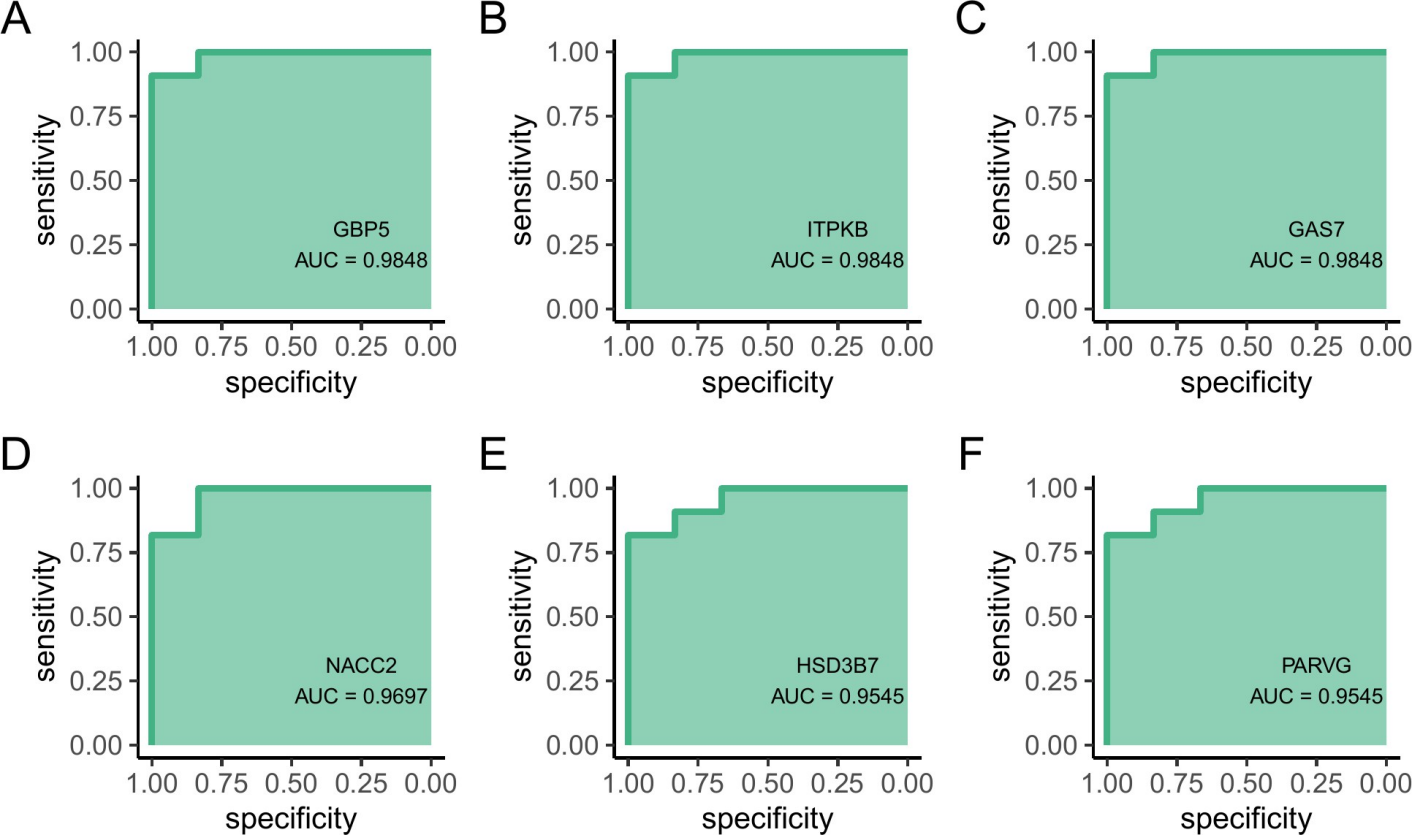
Receiver operating characteristic (ROC) curves for the hub genes. (A) *GBP5*, (B) *ITPKB*, (C) *GAS7I*, (D) *NACC2*, (E) *HSD3B7*, and (F) *PARVG*.

We used ROC curves to further validate the diagnostic value of the hub genes, revealing that the area under the ROC curve (AUC) values for *GBP5* (AUC = 0.985), *ITPKB* (AUC = 0.985), *GAS7* (AUC = 0.985), *NACC2* (AUC = 0.97), *HSD3B7* (AUC = 0.955), and *PARVG* (AUC = 0.955) were all greater than 0.6 (Fig 5(A)–5(F)). This suggests the potential of these hub genes for diagnosing MAB infection.

### 3.10 Signaling pathways related to hub genes

**Fig 6:**
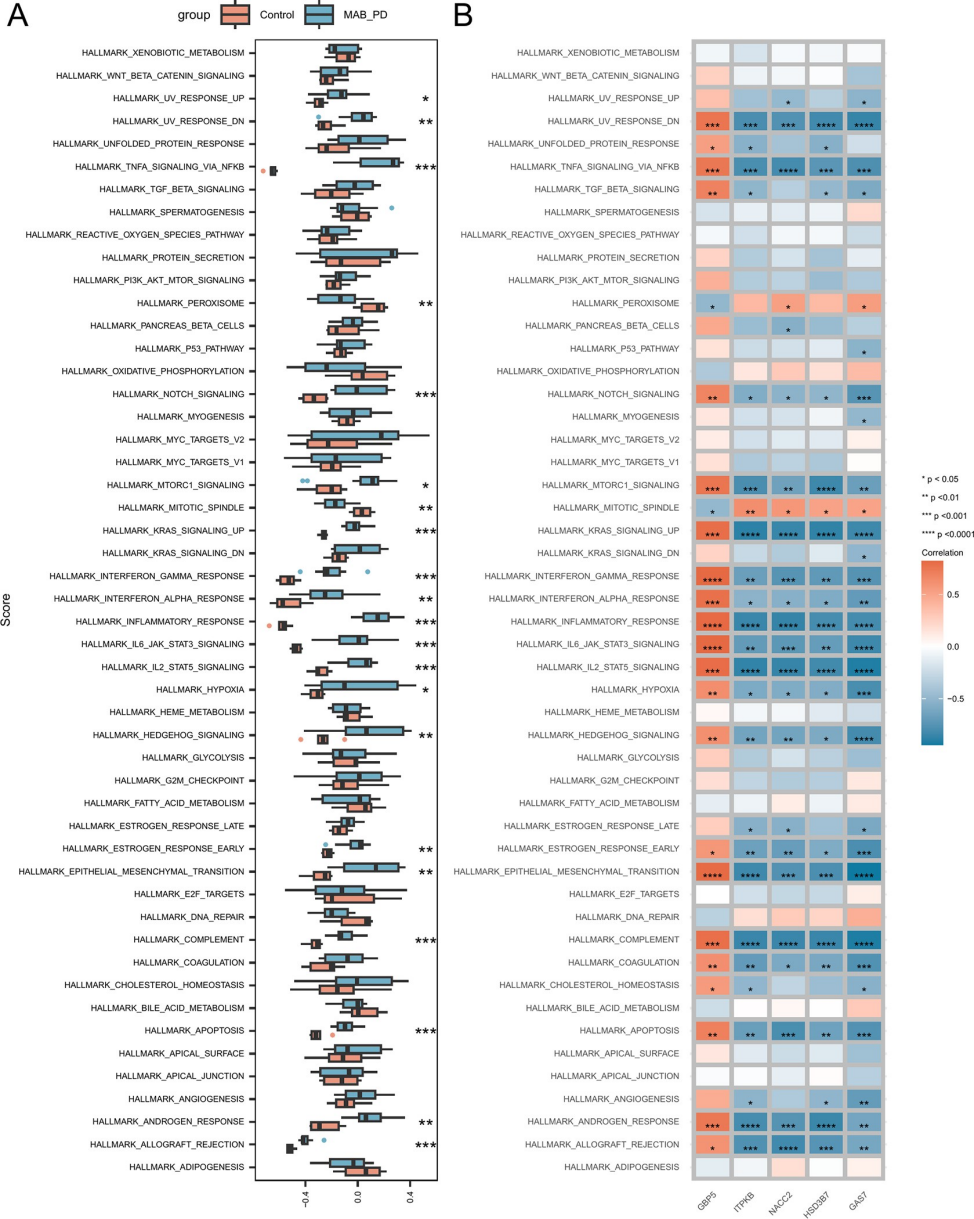
Correlation between hub genes and 50 HALLMARK signaling pathways. (A) Comparative analysis of 50 HALLMARK signaling pathways between the MAB_PD and control groups. (B) Correlation analysis of hub genes with 50 HALLMARK signaling pathways. Asterisks indicate p-values; ****p < 0.0001, ***p < 0.001, **p < 0.01, *p < 0.05.

Furthermore, GSVA was conducted to examine the differences in the 50 hallmark signaling pathways between THP-1-derived macrophages with MAB infection and the control group. In THP-1-derived macrophages infected with MAB, 19 hallmark signaling pathways were significantly upregulated, while two pathways were considered significantly downregulated, suggesting that these pathways play an important role in MAB infection (Fig 6(A), S10 Table). We also analyzed the correlation among the five DEGs, the most significant hub genes, and the 50 hallmark signaling pathways (Fig 6(B)).

### 3.11 Interaction network of hub genes with RBP, ceRNA, and TF

**Fig 7:**
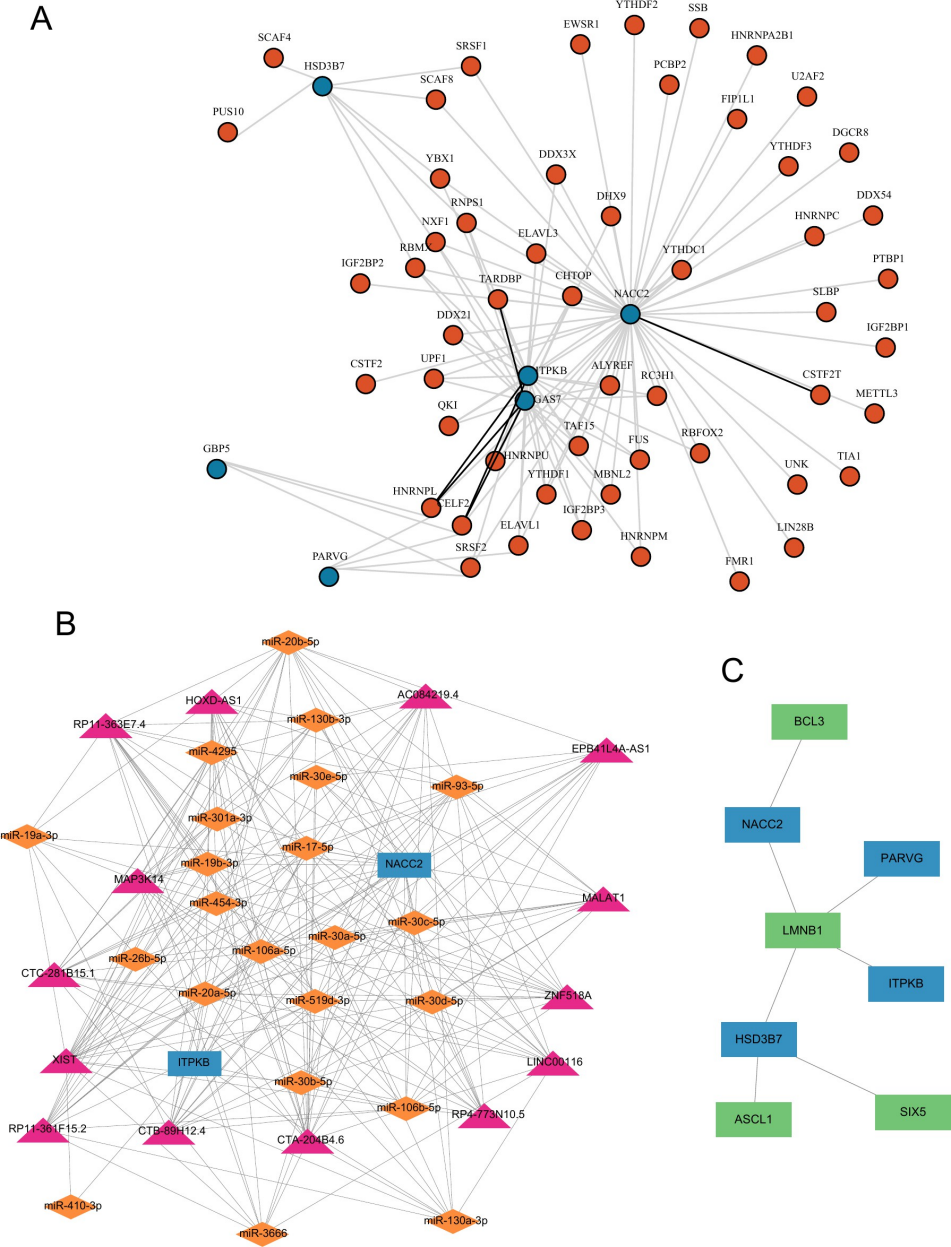
Interaction network of hub genes with RBP, ceRNA, and TF. (A) mRNA-RBP Interaction Network, where blue circles denote mRNAs of hub genes, and orange circles represent RBPs. (B) lncRNA-miRNA-mRNA Network for Hub Genes. Pink triangles symbolize lncRNAs, blue rectangles represent mRNAs of hub genes, and orange diamonds depict miRNAs. (C) TF-mRNA Interaction Network, with sky blue rectangles representing mRNAs of hub genes, and light green rectangles indicating TFs.

Given the role of RBPs in mRNA regulation, we utilized the StarBase online database to identify six hub genes and retrieved the corresponding mRNA/RBP pairs. Leveraging the dataset’s provided gene relationships, we constructed an RBP-mRNA network comprising 59 nodes: 53 RBPs, 6 mRNAs, and 113 edges. The six bold lines in the Fig signify the most significant mRNA-RBP interactions. Notably, hub gene *GAS7* displays robust interactions with RBPs such as CELF2, HNRNPL, and TARDBP. Similarly, hub gene *ITPKB* exhibits strong interactions with CELF2 and HNRNPL. Additionally, hub gene *NACC2* demonstrates a pronounced interaction with the RBP CSTF2T. For further insight, detailed information on the nodes and interactions is provided in S11 Table, while a visual representation of the network is presented in Fig 7(A).

To decipher the underlying molecular mechanisms of hub genes, we established a ceRNA regulatory network, specifically an interactive network of lncRNA–miRNA–mRNA. The network, visualized through Cytoscape, encompassed 2 mRNAs, 14 lncRNAs, and 22 miRNAs, resulting in a total of 38 nodes and 231 edges (Fig 7(B)).

To investigate TFs binding to hub genes, we explored the hTFtarget database, acquiring interaction data between four hub genes (*HSD3B7*, *NACC2*, *ITPKB*, *PARVG*) and four TFs. Utilizing Cytoscape software, we visualized these interactions (Fig 7(C)). Within the TF-mRNA interaction network, *HSD3B7* displayed the highest number of interactions with TFs, totaling three TF-mRNA pairs. Detailed TF-mRNA interactions are provided in S12 Table.

## 4 Discussion

This study aimed to identify the ferroptosis-related genes associated with MAB infection and explore potential biomarkers for this disease. GSEA and GSVA revealed that the genes were predominantly enriched in the Nod-like receptor (NLR) signaling pathway, oxidative phosphorylation, and peroxisome.

The NLR signaling pathway is crucial for detecting intracellular pathogens like MAB and triggering the innate immune response [29, 30]. NOD2, a critical receptor in this pathway, recognizes bacterial components and triggers downstream signaling, which leads to cytokine production and nitric oxide (NO) synthesis. This process enhances the immune response against MAB by boosting macrophage antibacterial activity [31–34]. NOD2 deficiency has been shown to impair macrophage control of MAB growth [35]. In our analysis, the enrichment of the NLR signaling pathway indicates an enhanced macrophage response to MAB infection. The activation of this pathway highlights its role in macrophage defense against MAB and suggests that targeting it could improve immune clearance.

Oxidative phosphorylation is a key metabolic pathway responsible for ATP production [36], and during MAB infection, this pathway is significantly upregulated in macrophages. This suggests an increased energy demand and heightened metabolic activity induced by the infection. Oxidative phosphorylation is linked to the production of reactive oxygen species (ROS), which macrophages use to kill intracellular pathogens [37]. However, MAB has evolved mechanisms to resist oxidative stress, allowing it to persist within macrophages [38]. The upregulation of this pathway in our study highlights the complex metabolic interactions between the host and the pathogen. Additionally, certain antibiotics, such as imipenem and cefoxitin, have been shown to induce oxidative phosphorylation bursts, enhancing their bactericidal activity against MAB [39].

We identified six ferroptosis-related DEGs in MAB infection: *GBP5*, *ITPKB*, *GAS7*, *NACC2*, *HSD3B7*, and *PARVG*. ROC curves confirmed their significant diagnostic value for MAB infection. This indicates that by detecting the abnormal expression of these genes in patients, it is possible to determine the occurrence of MAB infection and potentially use these genes as primary therapeutic targets, benefiting the patients. GO enrichment analysis indicated that ferroptosis-related DEGs influence metabolic reactions and energy conversion in MAB infection. Furthermore, GO analysis of the hub genes and their close interacting partners revealed their involvement in diverse biological processes and molecular functions, including immune responses, inflammatory reactions, cell metabolism, cell death, and apoptosis, underscoring the significance of these pathways in the development of MAB infection.

Through an analysis of hub gene expression patterns across distinct groups, we observed a significant upregulation of *GBP5* in both MAB_R and MAB_S compared to the control group. Notably, *GBP5*, recognized as an interferon-induced gene [40], holds prominence as an immune-related gene. Its heightened expression suggests an augmented immune response by macrophages during MAB infection, hinting at *GBP5*’s potential diagnostic utility for MAB infection.

GSVA showed significant upregulation of the apoptosis pathway in MAB-infected macrophages compared to controls. Additionally, the ferroptosis-related hub gene *GBP5* in MAB infection exhibited a positive correlation with the apoptosis signaling pathway. Clinical MAB strains with high virulence can induce significantly more cell apoptosis than non-virulent strains [41], which suggests that macrophage apoptosis can be suppressed by downregulating *GBP5*. Ferroptosis, an iron-dependent form of cell death, is distinct from apoptosis [10], but their signaling pathways may interact. Further research is needed to validate these connections.

MAB is recognized by innate immune receptors, triggering an inflammatory response. This response involves the production of cytokines such as TNF-α, IL-1β, and IL-6, which recruit immune cells like neutrophils and macrophages to the site of infection [42]. These cytokines coordinate immune defense by promoting phagocytosis and granuloma formation [43]. Despite these defense mechanisms, MAB is highly resistant to intracellular killing mechanisms, and its persistent presence within macrophages can lead to chronic inflammation [38]. Prolonged inflammation may result in tissue damage and the formation of fibrotic lesions in the lungs, thus promoting disease progression [44]. MAB’s ability to both promote inflammation and evade immune responses enables it to survive for extended periods within the host [5]. In the present study, GSVA revealed that the hub genes were primarily enriched in inflammation-related signaling pathways, such as inflammatory responses, the IL-2 STAT5 signaling pathway, and IL6 JAK STAT3 signaling. This suggests that these ferroptosis-related genes can activate inflammation-related signaling pathways to promote ferroptosis. Multiple studies have reported a close association between the activation of inflammation-related signaling pathways and ferroptosis [45–47]. Fig 6(B) shows that the ferroptosis-related hub gene *GBP5* in MAB infection is positively correlated with the above-mentioned inflammation-related signaling pathways, suggesting that macrophage ferroptosis can be suppressed by downregulating *GBP5*. Currently, research on inflammation in MAB infection is limited and requires further investigation.

Furthermore, GSVA revealed the significant upregulation of genes expressed by macrophages in the hypoxia pathway in macrophages with MAB infection. The HIF transcription factor is the key regulator of hypoxia. HIF-2 can increase the expression of *PLIN2* and *HILPDA*, thereby promoting lipid accumulation and oxidative stress and ultimately facilitating ferroptosis [48]. The ferroptosis-related hub gene *GBP5* was positively correlated with the hypoxia pathway in MAB infection, suggesting that downregulating *GBP5* may suppress macrophage ferroptosis. These results provide new insights into the mechanism of ferroptosis in MAB infection.

In this investigation, we probed the intricate interplay between RBPs and mRNAs, culminating in the establishment of an RBP-mRNA interaction network. Notably, hub genes such as *GAS7*, *ITPKB*, and *NACC2* displayed substantial interactions with multiple RBPs, including CELF2, HNRNPL, TARDBP, and CSTF2T. These RBPs typically oversee various pivotal functions, including RNA stabilization, splicing, transport, and translational regulation. The robust binding affinity between these RBPs and hub mRNAs underscores the intricate and finely tuned regulatory mechanisms governing the fate of these mRNAs within the cellular milieu.

Recent studies have highlighted the involvement of lncRNAs and miRNAs in the pathogenesis and host defense mechanisms of NTM infection [49, 50]. To clarify the interactions between lncRNAs, miRNAs, and ferroptosis-related genes, we constructed a ceRNA regulatory network. We predicted that 22 miRNAs regulate two key ferroptosis-related genes, *NACC2* and *ITPKB*. We also identified potential interactions between these 22 miRNAs and 14 lncRNAs. This discovery highlights the involvement of ferroptosis, mediated by *NACC2* and *ITPKB*, in the pathogenesis of MAB infection, warranting further exploration to uncover the underlying mechanisms.

In this study, we conducted an exhaustive analysis of TFs binding to hub genes using the hTFtarget database, successfully identifying interaction relationships between four hub genes (*HSD3B7*, *NACC2*, *ITPKB*, *PARVG*) and four TFs. Remarkably, the interaction involving the hub gene *HSD3B7* and three transcription factors (LMNB1, ASCL1, SIX5) was notably significant. LMNB1 and ASCL1 have been recognized as upstream transcription factors of ferroptosis-related biomarkers [51, 52]. Hence, we hypothesize that LMNB1 and ASCL1 may enhance the transcriptional activity of *HSD3B7*, thereby inducing ferroptosis in macrophages by modulating iron metabolism and ROS production, consequently fostering MAB infection occurrence.

Our study has some limitations. Firstly, although we identified six genes associated with ferroptosis in MAB-infected macrophages, further animal and cell experiments are needed to validate these findings, which we plan to conduct in future studies. Secondly, our analysis suggests that these genes may influence MAB infection by affecting pathways such as apoptosis, hypoxia, inflammation, and immune response. However, further research is required to clarify the precise roles of these genes in these pathways. Lastly, the diagnostic value of these genes needs to be confirmed in larger patient cohorts with MAB infection.

## 5 Conclusion

We used bioinformatic tools to identify ferroptosis-related genes for MAB infection. Our results suggest that these genes exhibit high diagnostic value for MAB infection. These findings not only reveal potential biomarkers for MAB infection but also enhance our comprehension of its pathogenesis.

## Supporting information

S1 Fig Gene expression differences in THP-1-derived macrophages with rough-type *M*. *abscessus* (MAB_R) infections. (A) Volcano plot illustrating the distribution of differentially expressed genes (DEGs) between the MAB_R and control samples. Red, blue, and gray dots represent the upregulated, downregulated, and non-significant DEGs, respectively. (B) The heatmap depicts the top 11 significantly upregulated and downregulated DEGs. (C) Boxplots depict the differences in the expression of genes between the MAB_R and control samples, with significance determined using the rank-sum test. Asterisks denote p-values; ****p < 0.0001, ***p < 0.001, **p < 0.01, *p < 0.05.
(TIF)

S2 Fig Pathways significantly enriched in THP-1-derived macrophages with rough-type *M*. *abscessus* (MAB_R) infections. Gene Set Enrichment Analysis (GSEA) revealed (A) chemokine signaling pathway, (B) Nod-like receptor signaling pathway, (C) JAK-STAT signaling pathway, (D) pentose and glucuronate interconversions, (E) drug metabolism cytochrome P450, and (F) retinol metabolism. (G) Visualization of gene set variation analysis (GSVA) through a heatmap.
(TIF)

S3 Fig Gene expression differences in THP-1-derived macrophages with smooth-type *M*. *abscessus* (MAB_S). (A) The volcano plot depicts the distribution of DEGs between the MAB_S and control group samples. Red, blue, and gray dots represent the upregulated, downregulated, and non-significant DEGs, respectively. (B) The heatmap illustrates the 13 significantly upregulated and downregulated DEGs. (C) The boxplot depicts the differential expression levels of genes between the MAB_S and control group samples, with statistical significance assessed using the rank-sum test. Asterisks indicate p-values; ****p < 0.0001, ***p < 0.001, **p < 0.01, *p < 0.05.
(TIF)

S4 Fig Pathways significantly enriched in THP-1-derived macrophages with smooth-type *M*. *abscessus* (MAB_S). GSEA analysis revealed (A) olfactory transduction, (B) Huntington’s disease, (C) oxidative phosphorylation, (D) peroxisome, (E) drug metabolism cytochrome P450, and (F) retinol metabolism. (G) Visualization of GSVA through a heatmap.
(TIF)

S5 Fig Interaction analysis of hub genes. (A) Gene co-expression network diagram. (B) Gene ontology (GO) analysis of co-expressed genes.
(TIF)

S1 Table Ferroptosis-related genes. (XLSX)

S2 Table Differentially expressed genes of MAB_R. (XLSX)

S3 Table Gene set enrichment analysis results for MAB_R. (XLSX)

S4 Table MAB_R gene set variation analysis enrichment results. (XLSX)

S5 Table Differentially expressed genes of MAB_S. (XLSX)

S6 Table Gene set enrichment analysis results for MAB_S. (XLSX)

S7 Table MAB_S gene set variation analysis enrichment results. (XLSX)

S8 Table Gene ontology pathway enrichment results. (XLSX)

S9 Table Gene ontology enrichment analysis results for protein-protein interaction network of genes. (XLSX)

S10 Table Hallmark enrichment results. (XLSX)

S11 Table Hub gene interactions with RNA binding protein. (XLSX)

S12 Table Transcription factor-mRNA interactions. (XLSX)
