# Supplementary figures and images for: Identification of biomarkers associated with ferroptosis in macrophages infected with Mycobacterium abscessus using bioinformatic tools
Source: PLoS One. 2025 Jan 10;20(1):e0314114. doi: 10.1371/journal.pone.0314114 (PMC11723624; doi:10.1371/journal.pone.0314114)

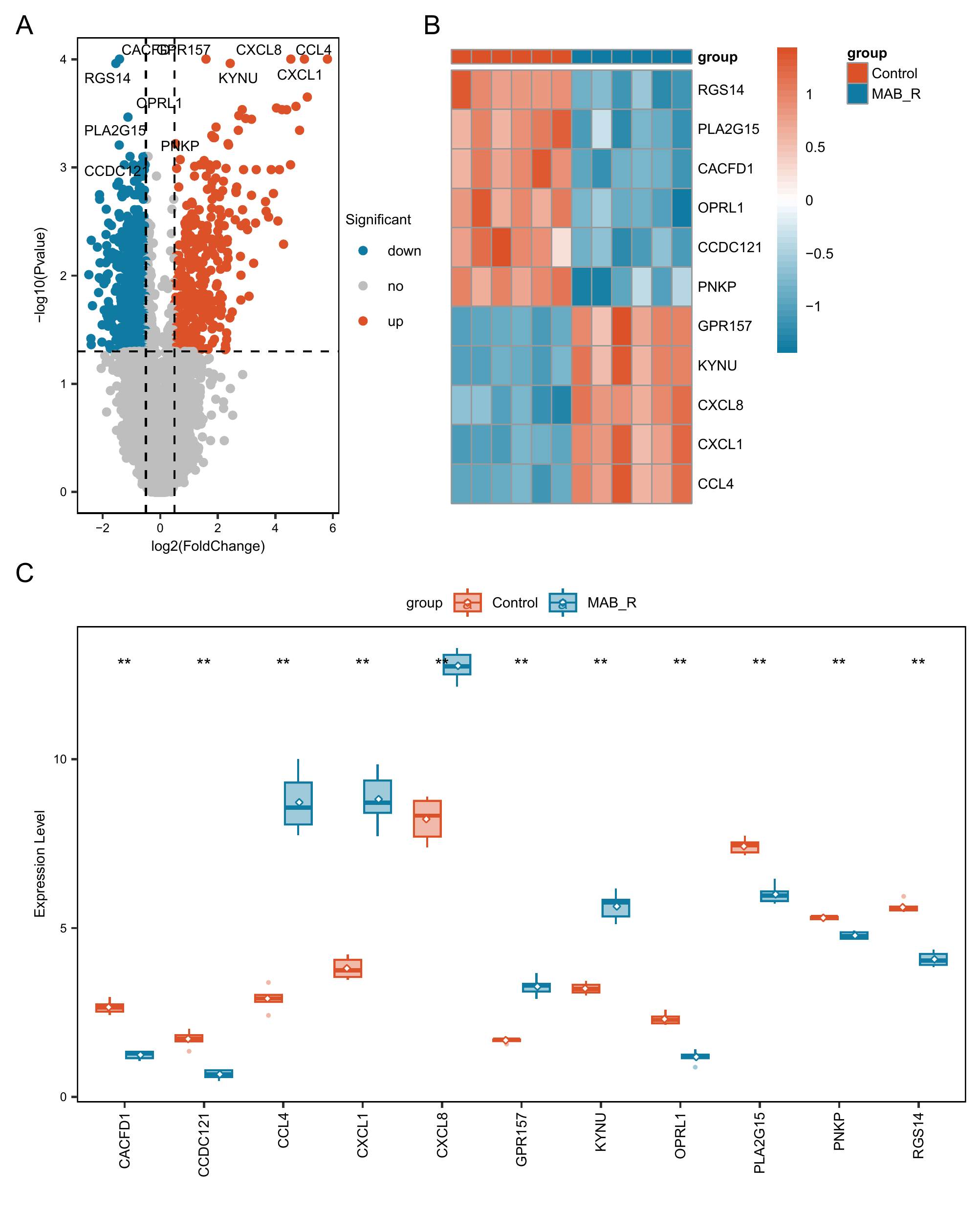

Supplement: S1 Fig — (A) Volcano plot illustrating the distribution of differentially expressed genes (DEGs) between the MAB_R and control samples. Red, blue, and gray dots represent the upregulated, downregulated, and non-significant DEGs, respectively. (B) The heatmap depicts the top 11 significantly upregulated and downregulated DEGs. (C) Boxplots depict the differences in the expression of genes between the MAB_R and control samples, with significance determined using the rank-sum test. Asterisks denote p-values; ****p < 0.0001, ***p < 0.001, **p < 0.01, *p < 0.05. (TIF) [file pone.0314114.s001.tif]

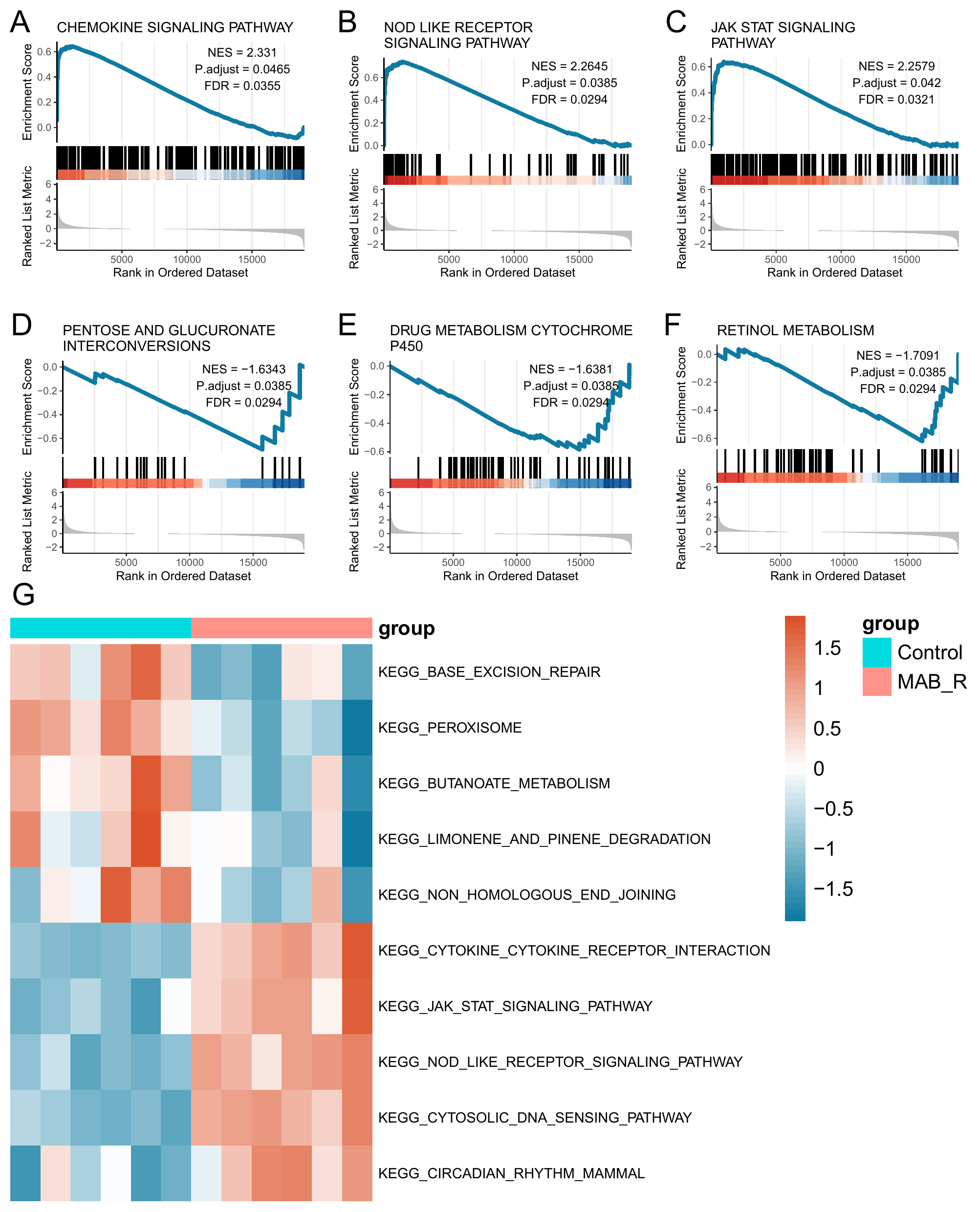

Supplement: S2 Fig — Gene Set Enrichment Analysis (GSEA) revealed (A) chemokine signaling pathway, (B) Nod-like receptor signaling pathway, (C) JAK-STAT signaling pathway, (D) pentose and glucuronate interconversions, (E) drug metabolism cytochrome P450, and (F) retinol metabolism. (G) Visualization of gene set variation analysis (GSVA) through a heatmap. (TIF) [file pone.0314114.s002.tif]

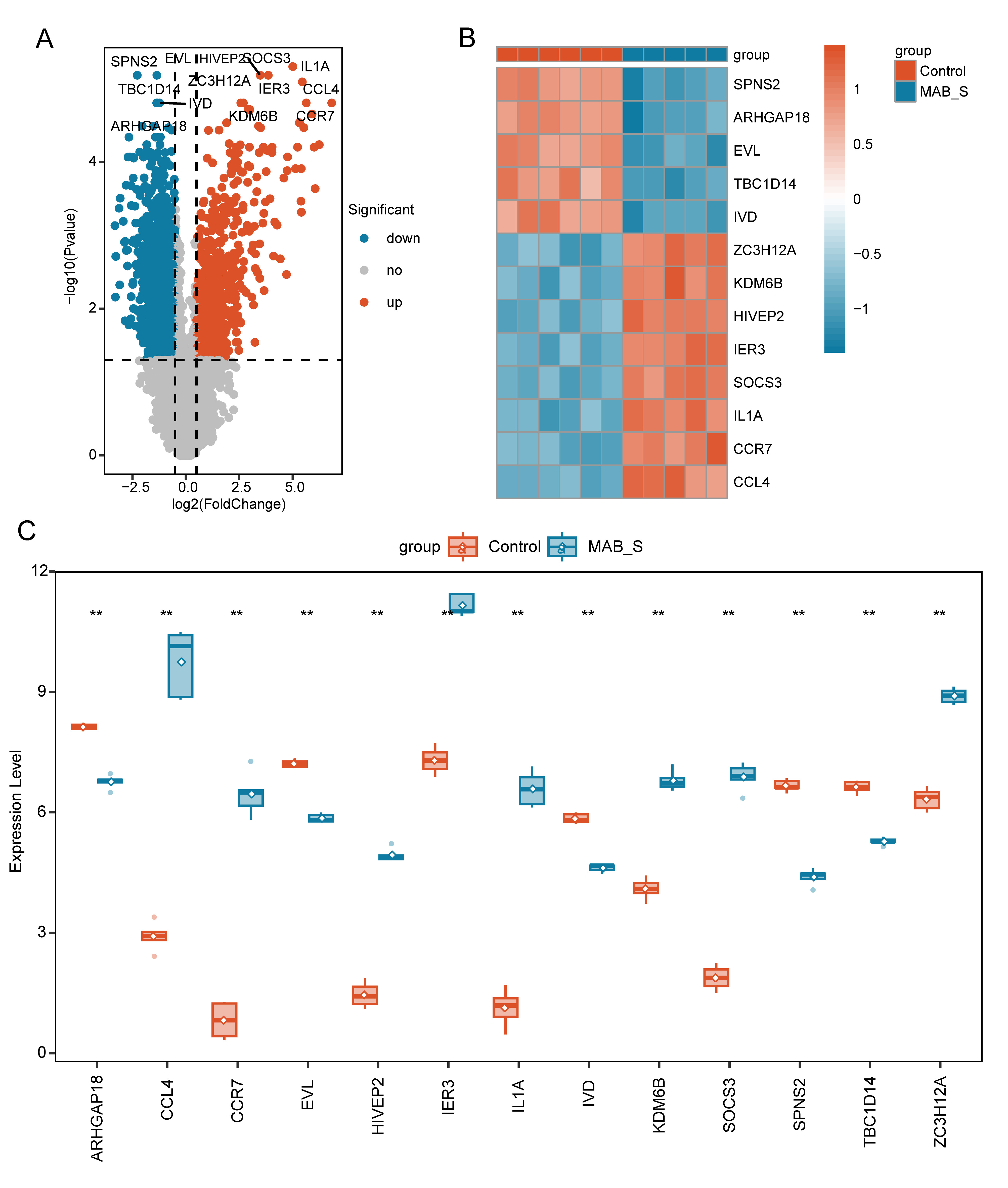

Supplement: S3 Fig — (A) The volcano plot depicts the distribution of DEGs between the MAB_S and control group samples. Red, blue, and gray dots represent the upregulated, downregulated, and non-significant DEGs, respectively. (B) The heatmap illustrates the 13 significantly upregulated and downregulated DEGs. (C) The boxplot depicts the differential expression levels of genes between the MAB_S and control group samples, with statistical significance assessed using the rank-sum test. Asterisks indicate p-values; ****p < 0.0001, ***p < 0.001, **p < 0.01, *p < 0.05. (TIF) [file pone.0314114.s003.tif]

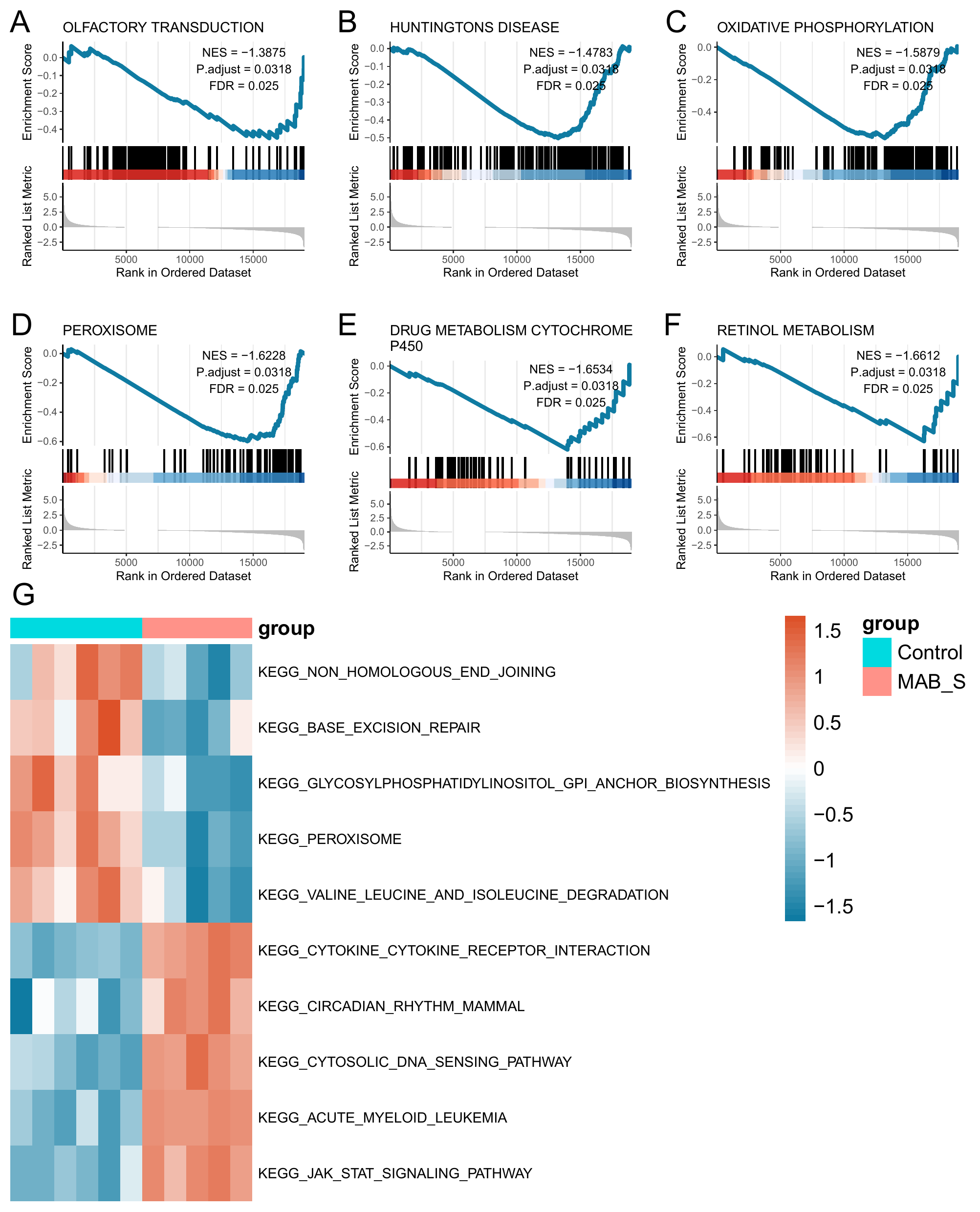

Supplement: S4 Fig — GSEA analysis revealed (A) olfactory transduction, (B) Huntington’s disease, (C) oxidative phosphorylation, (D) peroxisome, (E) drug metabolism cytochrome P450, and (F) retinol metabolism. (G) Visualization of GSVA through a heatmap. (TIF) [file pone.0314114.s004.tif]

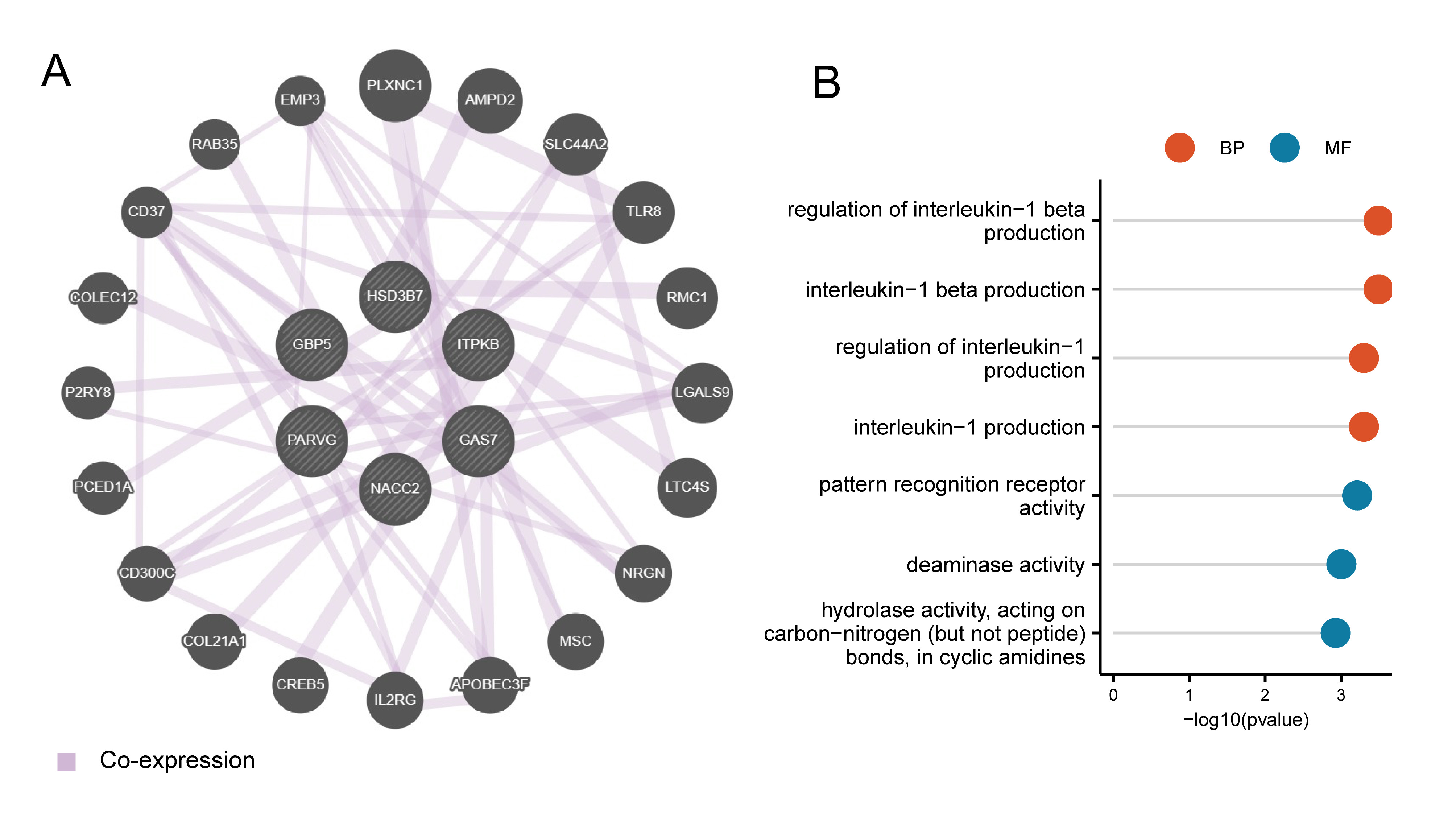

Supplement: S5 Fig — (A) Gene co-expression network diagram. (B) Gene ontology (GO) analysis of co-expressed genes. (TIF) [file pone.0314114.s005.tif]
